# Avaliação Macroscópica da Aterosclerose nas Artérias: Uma Ferramenta de Avaliação na Autópsia

**DOI:** 10.36660/abc.20190846

**Published:** 2021-06-08

**Authors:** Mariana Silva Oliveira, Bianca Gonçalves Silva Torquato, Maria Helena Soares, Maria Luiza Gonçalves dos Reis Monteiro, Guilherme Ribeiro Juliano, Laura Sanches Aguiar, Vicente de Paula Antunes Teixeira, Mara Lúcia da Fonseca Ferraz

**Affiliations:** 1 Universidade Federal do Triângulo Mineiro UberabaMG Brasil Universidade Federal do Triângulo Mineiro , Uberaba , MG – Brasil

**Keywords:** Doenças Cardiovasculares, Aterosclerose, Fatores de Risco, Doenças Assintomáticas, Artérias, Autópsia, Hereditariedade, Diagnóstico Precoce

## Abstract

**Fundamento:**

A aterosclerose, em alguns casos, é uma condição assintomática, sendo necessário conhecer o grau de comprometimento arterial provocado pelas placas e sua associação com os fatores de risco. O exame de autópsia permite a compreensão dos processos básicos de doenças, assim como a avaliação e fornecimento de dados sobre a característica macroscópica do acometimento aterosclerótico.

**Objetivo:**

Avaliar macroscopicamente e padronizar o acometimento aterosclerótico das artérias aorta, carótidas e ilíacas e comparar com a idade, o sexo e a causa de morte.

**Métodos:**

Foram coletados 53 artérias aorta, 53 artérias carótida direita, 53 artérias carótida esquerda, 53 artérias ilíaca direita e 53 artérias ilíaca esquerda. Para essa avaliação, foi considerada a extensão de estrias lipídicas, de placas ateromatosas, de fibrose e de calcificação, as quais serviram de referência para pontuar a intensidade do acometimento aterosclerótico. Foram observados vários graus da aterosclerose e valores acurados para a classificação discreta, moderada e acentuada. Para a análise estatística, os dados foram analisados utilizando-se o software *GraphPad Prism* ^®^ 7.0. As diferenças foram consideradas estatisticamente significativas quando “p” foi menor que 5% (p<0,05).

**Resultados:**

As artérias carótidas apresentaram maior acometimento aterosclerótico em comparação às outras artérias avaliadas (K=15,73, p=0,0004). A ocorrência da aterosclerose se mostrou progressiva e significativa com o decorrer da idade (carótidas: t=6,321; p<0,0001; aortas: U=83,5; p<0,0001; ilíacas: U=306; p<0,0001) e na causa de morte cardiovascular (carótidas: t=5,047; p<0,0001; aortas: U=98,5; p=0,0068; ilíacas: U=467,5; p=0,0012).

**Conclusão:**

A avaliação macroscópica da aterosclerose trata-se de uma forma inovadora e de baixo custo de avaliação através da visualização direta das placas ateroscleróticas, possibilitando uma associação com fatores de risco como idade avançada e doenças cardiovasculares, fornecendo dados importantes para a prática clínica.

## Introdução

A aterosclerose é uma doença multifatorial associada a fatores hereditários, sexo e hábitos de vida como o tabagismo, alimentação inadequada e pouca ou nenhuma atividade física. ^1^ A presença e progressão da placa aterosclerótica provoca inflamação e deposição de lipídios nas paredes das artérias ^1^ que podem desencadear o desenvolvimento de doenças cardiovasculares com alta incidência a nível mundial. ^2^

O diagnóstico precoce da aterosclerose como preditor da doença arterial coronariana e do infarto agudo do miocárdio se faz necessário para reduzir a morbidade e mortalidade associada à doença. Estudos exploram a prevalência e a associação de fatores que contribuem para estratificação dos riscos. ^3^ A avaliação e o diagnóstico precoces em pacientes que se enquadram nesses grupos se fazem importantes.

Sabe-se que o exame de autópsia é de extrema importância, pois permite a compreensão dos processos básicos das doenças. ^4^ Estudos têm demonstrado que além da eliminação dos fatores de risco associados à aterosclerose existem medicamentos atualmente eficazes para o tratamento dessa doença. ^5^ No entanto, para a eficácia do tratamento é necessário conhecer o grau de comprometimento arterial provocado pelas placas ateroscleróticas. ^6^ Tal fato torna a nossa avaliação e fornecimento de dados sobre a característica macroscópica ainda mais valiosa, pois estudos de autópsia permitem uma visualização ampla e direta da aterosclerose. ^7 , 8^

Por se tratar de uma doença cardiovascular, multifatorial e responsável pelo desenvolvimento de enfermidades graves, a avaliação macroscópica da aterosclerose em material de autópsia é importante, pois fornece uma descrição fidedigna e padronizada sobre a progressão da placa aterosclerótica. A associação dos aspectos macroscópicos com os fatores de risco contribue para o fornecimento de dados epidemiológicos para a clínica. O objetivo do trabalho foi identificar macroscopicamente a intensidade do acometimento aterosclerótico das artérias aorta, carótidas e ilíacas e comparar o grau do acometimento nesses leitos arteriais e com fatores de risco como a idade, o sexo e a causa de morte.

## Métodos

Foram avaliados 2.931 protocolos de autópsias realizadas, no período de 1963 a 2018. A partir desses protocolos, foram selecionados os materiais biológicos (artérias carótidas, aorta e ilíacas) daqueles pacientes que apresentaram o laudo de autópsia completo, com informações relativas à idade (selecionados com idade superior a 18 anos), ao sexo e a causa de morte (cardiovascular ou não). Os casos em que os materiais biológicos não se encontravam em bom estado de conservação ou que tinham o laudo de autópsia incompleto foram excluídos do estudo. Foi obtida uma amostra de 53 pacientes autopsiados. Após a seleção da amostra, do arquivo de peças anatômicas da disciplina de Patologia Geral, foram coletados 53 artérias aorta, 53 artérias carótida direita, 53 artérias carótida esquerda, 53 artérias ilíaca direita e 53 artérias ilíaca esquerda.

O estudo foi desenvolvido na disciplina de Patologia Geral da Universidade Federal do Triângulo Mineiro e aprovado pelo Comitê de Ética em Pesquisa da Universidade Federal do Triângulo Mineiro sob o número de Certificado de Apresentação de Apreciação Ética (CAAE): 56931816.4.0000.5154 em conformidade com a resolução 466/2012.

### Avaliação macroscópica da aterosclerose

**Figura 1:**
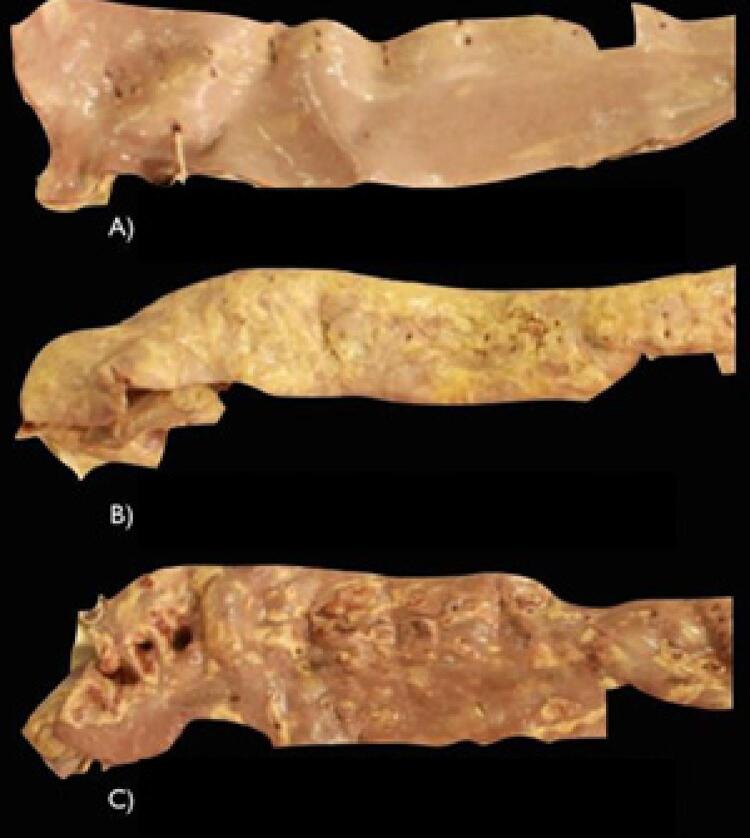
– A) Artéria aorta com estrias lipídicas. B) Artéria aorta com placas ateromatosas. C) Artéria aorta com placas ateromatosas, fibrose e calcificações.

**Figura 2:**
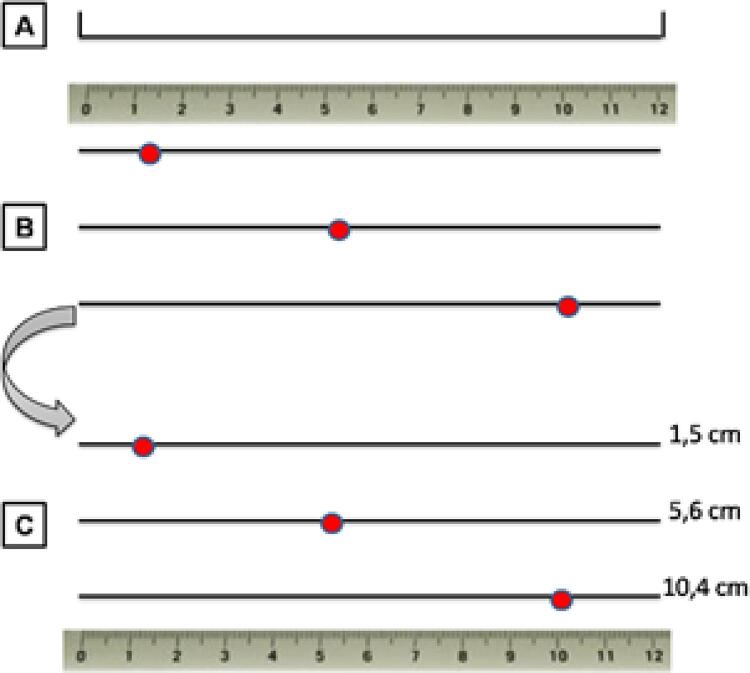
– A) Modelo de linha de 12,0 cm não milimetrada utilizada como escala para o acometimento da aterosclerose. B) Registro do ponto na escala não milimetrada, referente à intensidade do acometimento, após avaliação das lesões. C) Medida da distância do ponto 0,0 cm ao ponto marcado após a finalização das avaliações em todas as artérias.

Três examinadores realizaram a classificação da intensidade macroscópica da aterosclerose nas artérias aorta, carótida direita, carótida esquerda, ilíaca direita e ilíaca esquerda. Foi feita utilizando-se um critério já descrito na literatura. ^9^ A progressão de estrias lipídicas, de placas ateromatosas, de fibrose e de calcificação, na parede das artérias serviram de referência para pontuar a intensidade do acometimento ( Figura 1 ). Em uma folha foi feita uma linha de 12,0 cm não milimetrada ( Figura 2A ). Essa linha foi utilizada como escala para o acometimento da aterosclerose.

Ao abrirem uma artéria, os examinadores observavam a progressão das lesões, em seguida, registrava-se um ponto na escala referente a intensidade do acometimento, quanto mais próximo do 0,0 cm menor o acometimento, e quanto mais próximo de 12,0 cm maior o acometimento ( Figura 2B ).

Após o final de todas as avaliações, foi realizada a medida da distância do ponto 0,0 cm ao ponto marcado na escala pelos examinadores, com a finalidade de evitar interferências nas classificações ( Figura 2C ). Para a classificação em discreto, moderado e acentuado, foram padronizadas medidas na escala. A intensidade da aterosclerose foi classificada como discreta quando a avaliação foi de 0,1cm a 4,0cm; moderada, de 4,1cm a 7,0cm e acentuada, de 7,1cm a 12,0cm. ^9^

**Figura 3:**
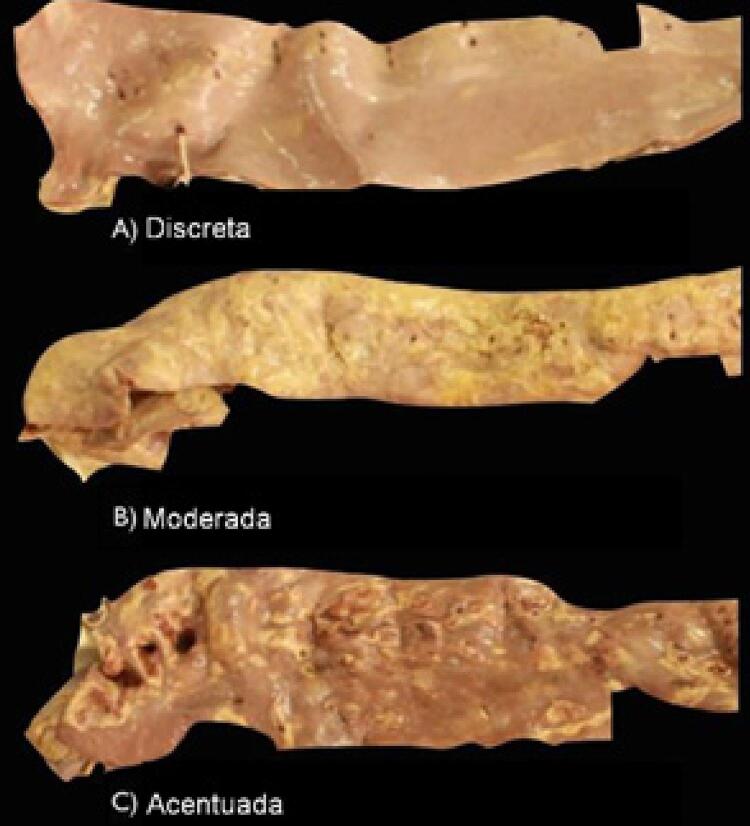
– A) Artéria aorta avaliada como discreta. B) Artéria aorta avaliada como moderada. C) Artéria aorta avaliada como acentuada.

Foram observados vários graus da aterosclerose e valores acurados para a classificação discreta, moderada e acentuada ( Figura 3 ).

### Análise estatística

Para a análise estatística foi elaborado um banco de dados no programa *Microsoft Excel* ^®^ . Os dados foram analisados utilizando-se o *software GraphPad Prism* ^*®*^ 7.0. Para verificar o tipo de distribuição das variáveis foi aplicado o teste estatístico de *Kolmogorov-Smirnov* (com *Dallal-Wilkinson-Lillie* para valor de p). Para as variáveis contínuas com distribuição normal foram apresentadas a média e desvio padrão e para as com distribuição não normal, a mediana e intervalo interquartil. Utilizamos o teste t de *student* (t) não pareado para a distribuição normal e o teste de *Mann-Whitney* (U) para a distribuição não normal na comparação de dois grupos. Para a comparação de três grupos, foi utilizado o teste de Kruskal-Wallis (H), seguido pelo pós-teste de Dunn. Para correlação foi empregado o coeficiente de correlação de *Spearman* (rS) para distribuição não-normal. As diferenças foram consideradas estatisticamente significativas quando “p” foi menor que 5% (p<0,05).

## Resultados

Com relação à distribuição geral da amostra, os dados estão descritos na Tabela 1 .

**Tabela 1:** – Características gerais da amostra

Variáveis	n (%)	Idade média± DP (anos)
**Total da amostra**	53 (100%)	49,9±18,6
**Idade**		
Idoso	17 (32,1%)	72,88±8,1
Não idoso	36 (67,9%)	39,05±10,24
**Sexo**		
Masculino total	26 (49,1%)	49,77±16,40
Feminino total	27 (50,9%)	50,04±20,77
Masculino idoso	7 (41,18%)	72±6,35
Feminino idoso	10 (58,82%)	74±9,42
Masculino não idoso	19 (52,78%)	41,58±9,84
Feminino não idoso	17 (47,22%)	36,24±10,22
**Causa de morte**		
Cardiovascular	10 (18,9%)	64,3±17,09
Não cardiovascular	43 (81,1%)	46,56±17,43

*n: amostra; DP: desvio padrão.*

As artérias carótidas apresentaram maior acometimento aterosclerótico em comparação às outras artérias avaliadas (H=15,73, p=0,0004), sendo encontrada diferença significativa entre as artérias carótidas e ilíacas (p=0,0002).

A variação da intensidade macroscópica da aterosclerose nas artérias analisadas está descrita na Tabela 2 .

**Tabela 2:** – Avaliação macroscópica das carótidas direita e esquerda, aortas e ilíacas direita e esquerda dos pacientes autopsiados

Variáveis	Carótida (cm)	Aorta (cm)	Ilíaca (cm)
**Idade**			
Idoso	7,256±2,254	8,6 (4,6-9,8)	5,9 (3,85-9,07)
Não idoso	3,983±2,59	1,8 (1,13-3,6)	1,15 (0,5-2,5)
	t=6,321; p<0,0001	U=83,5; p<0,0001	U=306; p<0,0001
**Sexo**			
Masculino	4,675±2,593	2,6 (1,28-6,18)	1,85 (0,6-4,78)
Feminino	5,378±3,178	3,7 (1,5-9)	2,85 (0,98-8,1)
	t=1,245; p=0,2160	U=285; p=2442	U=1165; p=0,1316
**Causa de morte**			
Cardiovascular	7,7±2,617	7,25 (3,33-10,58)	5,1 (2,65-7,18)
Não cardiovascular	4,413±2,625	2,2 (1,2-7)	1,8 (0,6-4,73)
	t=5,047; p<0,0001	U=98,5; p=0,0068	U=467,5; p=0,0012

*n:amostra; cm: centímetros.*

**Figura 4:**
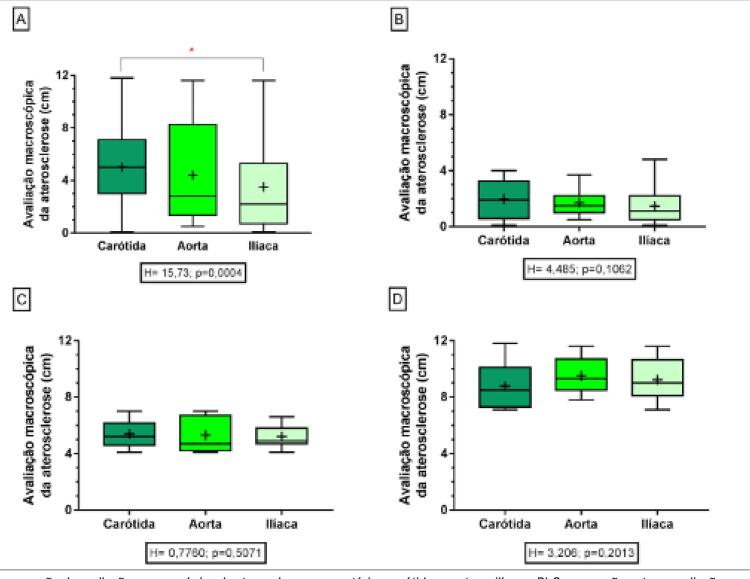
– A) Comparação da avaliação macroscópica da aterosclerose nas artérias carótidas, aortas e ilíacas. B) Comparação entre a avaliação macroscópica da aterosclerose nas artérias carótidas, aortas e ilíacas classificadas como discretas (0 a 4 cm). C) Comparação entre a avaliação macroscópica da aterosclerose nas artérias carótidas, aortas e ilíacas classificadas como moderadas (4,1 a 7 cm). D) Comparação entre a avaliação macroscópica da aterosclerose nas artérias carótidas, aortas e ilíacas classificadas como acentuadas (7,1 a 12 cm).

As distribuições da avaliação macroscópica da aterosclerose nas artérias carótidas, aortas e ilíacas e os diferentes graus do acometimento macroscópico da aterosclerose estão representados na Figura 4 .

**Figura 5:**
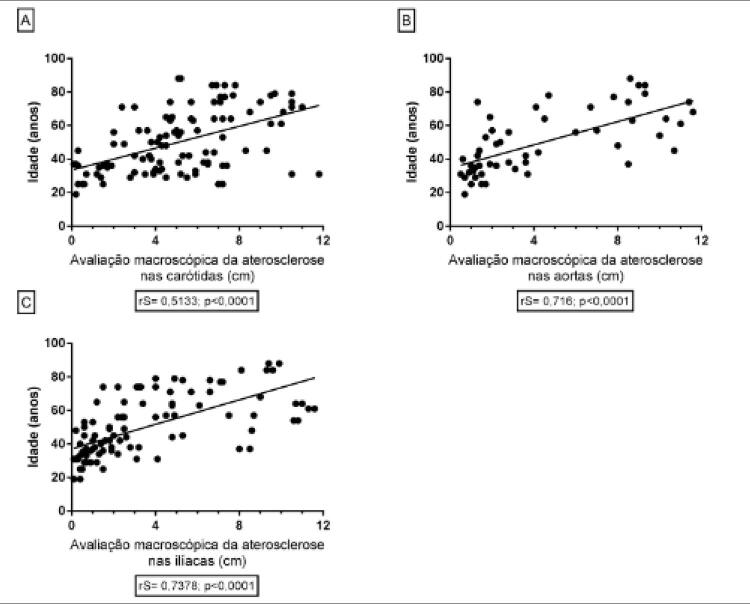
– A) Correlação entre a avaliação macroscópica da aterosclerose nas carótidas com a idade. B) Correlação entre a avaliação macroscópica da aterosclerose nas aortas com a idade. C) Correlação entre a avaliação macroscópica da aterosclerose nas ilíacas com a idade.

A ocorrência da aterosclerose se mostrou progressiva e significativa com o decorrer da idade nas carótidas (rS=0,5133; p<0,0001), nas aortas (rS=0,716; p<0,0001) e nas ilíacas (rS= 0,7378; p<0,0001) ( Figura 5 ).

De acordo com a avaliação macroscópica da aterosclerose com as variáveis analisadas, os dados estão descritos na Tabela 2 .

## Discussão

Por meio de uma amostra proveniente de material de autópsia, evidenciou-se o acometimento da aterosclerose em diferentes leitos arteriais sendo eles de grande importância para o suprimento sanguíneo do organismo. A análise macroscópica fornece um meio de compreender o processo de desenvolvimento da doença, sendo um instrumento válido e necessário para a pesquisa e fornecimento de dados para a clínica como já demonstrado em outros estudos. ^9 - 11^

O presente estudo demonstrou um maior acometimento nas artérias carótidas, quando comparada às artérias aorta e ilíacas, porém com diferença significativa somente entre as carótidas e ilíacas. Embora a aterosclerose seja um processo que possa acometer toda a árvore circulatória, sendo encontrada em qualquer artéria de grande ou médio calibre, a doença tende a localizar-se em áreas particulares do sistema arterial, como os segmentos aortoilíaco, ilíacofemoral ou as artérias carótidas. Fatores como modificações do fluxo sanguíneo, alterações da pressão extra vascular e particularidades anatômicas e bioquímicas parecem explicar a preferência das lesões por esses vasos. ^12^ Além disso a carótida é uma artéria avaliada em vários outros estudos como marcadora de aterosclerose e determinante para o desenvolvimento de doenças cardiovasculares. ^13 - 15^

De modo geral, a intensidade da aterosclerose variou entre discreta e moderada. Nas lesões avaliadas como discretas havia presença de estrias lipídicas, o que indica o início do processo da lesão. Embora essas lesões não alterem a circulação sanguínea por não obstruírem a luz vascular, a sua localização facilita a contínua deposição lipídica e evolução para aterosclerose. ^16^

Nos pacientes idosos houve uma intensidade maior e significativa da aterosclerose quando comparado aos não idosos. A idade tem sido demonstrada com um preditor significativo para o desenvolvimento da aterosclerose. ^17 , 18^ Foi encontrado um aumento de placas ateroscleróticas acentuadas e assintomáticas em artérias de pacientes idosos, ^19^ assim como calcificações, ^20^ o que corrobora com nossos achados.

Com relação ao sexo, foi encontrado maior intensidade da aterosclerose nas mulheres, porém sem diferença significativa, o que corrobora com um estudo semelhante que analisou a aterosclerose por meio de ultrassonografia, onde os autores não encontraram diferenças entre os sexos. ^21^ Um estudo recente comprovou que os sexos respondem fisiologicamente aos fatores de risco (tabagismo, obesidade, diabetes e hipertensão arterial sistêmica) de forma diferente, sendo o sexo feminino o mais acometido e sensibilizado frente às agressões citadas. Embora muitos estudos comprovem que maiores índices de eventos cardiovasculares são nos homens, vêm sendo apresentadas pesquisas divergentes a esse fato, visto que a resposta fisiológica do sexo feminino é mais sensível frente aos fatores de risco, o que contribui para o desenvolvimento ou piora do quadro da doença cardiovascular. ^22^

No presente estudo os pacientes que foram a óbito devido a causas cardiovasculares apresentaram intensidade significativamente maior de aterosclerose. O estudo anatomopatológico de pacientes que foram à óbito por causas cardiovasculares fornece a melhor amostra da população para se estudar a aterosclerose. ^4^ Doenças cardiovasculares estão diretamente associadas com a ocorrência da aterosclerose sistêmica, que na maioria das vezes é assintomática, ^23^ o que dificulta a prevenção, porém de extrema importância.

De forma geral, nosso estudo possui algumas limitações por ser um estudo post mortem, como a perda de algumas informações sobre os hábitos de vida anteriores dos pacientes como uso de medicações, alimentação, tabagismo, entre outros fatores de risco que também estão relacionados com o desenvolvimento da aterosclerose. Além disso, alguns óbitos ocorreram sem que a aterosclerose fosse investigada anteriormente durante a internação do paciente, o que seria um bom preditor para a acurácia da avaliação macroscópica. Porém existem vários pontos positivos que fortalecem o trabalho, como o fato da avaliação macroscópica direta e precisa através da visualização da placa em sua totalidade, e a confirmação da associação de fatores de risco intrínsecos como a idade e o sexo e também a causa de morte que pode ter ocorrido devido a fatores extrínsecos. Além disso, foram coletados vários leitos arteriais de importância para a circulação corpórea (carótida direita e esquerda, aorta e ilíaca direita e esquerda) que demonstraram resultados semelhantes quando comparados aos fatores de risco e que confirmaram serem bons locais para a avaliação da aterosclerose sistêmica.

## Conclusões

A aterosclerose apresenta um padrão de lesão progressivo ao longo da vida, que afeta diferentes leitos arteriais, sendo que as artérias carótidas são as mais acometidas, mostrando-se bons marcadores para estudo e avaliação da progressão da placa aterosclerótica. O estudo mostra a importância da avaliação da aterosclerose e traz uma forma inovadora de avaliação, pois é possível mensurar a intensidade macroscópica do acometimento através da visualização direta das placas ateroscleróticas e comparar com fatores de risco que, em associação podem contribuir para a progressão da placa e para o desenvolvimento de outras doenças cardiovasculares. A idade avançada, o sexo feminino e a causa de morte cardiovascular contribuem como fatores de risco para maior acúmulo lipídico nestas artérias. A avaliação macroscópica é um método de baixo custo, eficaz e padronizado para a mensuração da intensidade da aterosclerose e permite a melhor compreensão do desenvolvimento de outros eventos cardiovasculares no momento da autópsia, além de fornecer dados para a prática clínica.
