# Supplementary material for: Avaliação Macroscópica da Aterosclerose nas Artérias: Uma Ferramenta de Avaliação na Autópsia
Source: Arq Bras Cardiol. 2021 Jun 8;116(6):1119–26. [Article in Portuguese] doi: 10.36660/abc.20190846 (PMC8288540; doi:10.36660/abc.20190846)
Supplement: Translation

# Translation: Macroscopic Evaluation of Atherosclerosis in the Arteries: An Autopsy Assessment Tool

**Authors:** Mariana Silva Oliveira, Bianca Gonçalves Silva Torquato, Maria Helena Soares, Maria Luiza Gonçalves dos Reis Monteiro, Guilherme Ribeiro Juliano, Laura Sanches Aguiar, Vicente de Paula Antunes Teixeira, Mara Lúcia da Fonseca Ferraz

**Affiliations:** 1 Brazil Universidade Federal do Triângulo Mineiro, Uberaba, MG – Brazil

**Keywords:** Cardiovascular Diseases, Atherosclerosis, Risk Factors, Asymptomatic Disease, Arteries, Autopsy, Heredity, Early Diagnosis

## Abstract

**Background:**

Atherosclerosis, in some cases, is an asymptomatic condition, and it is important to know the degree of arterial impairment caused by plaques and its association with risk factors. Autopsy examination provides understanding of basic disease processes and assessment to data about macroscopic characteristic of atherosclerotic involvement.

**Objective:**

To macroscopically assess and standardize atherosclerotic involvement of aorta, carotid and iliac arteries and compare with age, gender and causes of death.

**Methods:**

We collected 53 aortic arteries, 53 right carotid arteries, 53 left carotid arteries, 53 right iliac arteries and 53 left iliac arteries. For this assessment, the extension of fatty streaks, atheromatous plaques, fibrosis and calcification were considered, being the reference to score the degree of atherosclerotic involvement. Many degrees of atherosclerosis and accurate values were observed for mild, moderate and severe classification. For statistical analysis, data were analyzed using the software GraphPad Prism® 7.0. Differences were considered statistically significant if p-value was less than 5% (p <0.05).

**Results:**

Carotid arteries had greater atherosclerotic involvement compared to the other arteries (K = 15.73, p = 0.0004). Atherosclerosis was progressive and significant with increasing age (carotid arteries: t = 6.321; p <0.0001; aorta: U = 83.5; p <0.0001; iliac: U = 306; p <0.0001) and as cause of cardiovascular death (carotids: t = 5.047; p <0.0001; aorta: U = 98.5; p = 0.0068; iliac: U = 467.5; p = 0.0012).

**Conclusion:**

Macroscopic assessment of atherosclerosis is an innovative and low-cost way of direct visualization of atherosclerotic plaques, enabling an association with risk factors such as increasing age and cardiovascular diseases, providing important data for clinical practice.

## Introduction

Atherosclerosis is a multifactorial disease associated with hereditary factors, sex and lifestyle habits such as smoking, inadequate diet and sedentary lifestyle.^1^ Inflammation and deposition of lipids in the artery wall are involved in triggering and progression of atherosclerotic plaques^1^ leading to cardiovascular diseases with high incidence worldwide.^2^

The early diagnosis of atherosclerosis as a predictor of coronary artery disease and acute myocardial infarction reduces morbidity and mortality associated with the disease. Studies explore prevalence and association of factors that contribute to risk stratification.^3^ Evaluation and early diagnosis in patients of these groups are important.

It is known that the autopsy exam is extremely important, as it allows the understanding of the basic processes of diseases.^4^ Studies have shown that in addition to eliminating risk factors associated with atherosclerosis, there are currently effective drugs for the treatment of this disease.^5^ However, for the treatment to be effective, it is worthy to know the degree of arterial impairment caused by atherosclerotic plaques.^6^ This fact makes our assessment and data on the macroscopic characteristics even more valuable, as autopsy studies allow a broad and direct view of atherosclerosis.^7 , 8^

As atherosclerosis is a multifactorial cardiovascular disease, responsible for the development of serious illnesses, its macroscopic assessment in autopsy material is important to provide a reliable and standardized description of atherosclerotic plaque progression. The association of macroscopic aspects with risk factors provides epidemiological data for clinical practice. The objective of this study was to identify macroscopically the intensity of atherosclerotic involvement of aorta, carotid and iliac arteries and to compare the degree of involvement with risk factors such as age, gender and cause of death.

## Methods

We evaluated 2931 autopsy protocols performed between 1963 and 2018. Based on these protocols, biological materials (carotid, aorta and iliac arteries) were selected from those patients who presented complete autopsy report, with information about age (included those over 18 years old), gender and the cause of death (cardiovascular or not). Cases with biological materials in inadequate conditions or with incomplete autopsy reports were excluded from the study. A sample of 53 autopsied patients was obtained from the anatomical specimen file of the Discipline of General Pathology, totalizing 53 aortic arteries, 53 right carotid arteries, 53 left carotid arteries, 53 right iliac arteries and 53 left iliac arteries. The study was developed in the Discipline of General Pathology of Federal University of Triângulo Mineiro and approved by the Research Ethics Committee of Federal University of Triângulo Mineiro with the Certificate of Presentation of Ethical Appreciation (CAAE) number: 56931816.4.0000.5154 in accordance with the resolution 466/2012.

### Macroscopic evaluation of atherosclerosis

Three examiners evaluated macroscopic degree of atherosclerosis in aorta, right carotid, left carotid, right iliac and left iliac arteries, using criteria already described in the literature.^9^ The progression of fatty streaks, atheromatous plaques, fibrosis and calcification on artery walls were a reference to score the degree of involvement ( Figure 1 ). A 12.0 cm non-millimeter line was made on a sheet ( Figure 2A ) and this line was used as a scale for atherosclerosis involvement.

**Figure 1:** – A) Aortic artery with fatty streaks. B Aorta with atheromatous plaques. C) Aorta with atheromatous plaques, fibrosis and calcifications.

**Figure 2:** – A) A 12.0 cm non-millimeter line model used as a scale for measurement of atherosclerosis degree. B) Registration of the point on the non-millimeter scale, referring to the degree of involvement after evaluation of lesions. C) Measure of the distance from the 0.0 cm point to the marked point after finishing evaluations in all arteries.

After opening the artery, examiners observed the degree of lesions, then a point was recorded on the scale regarding the degree of involvement, the closer to 0.0 cm the lesser the involvement, and the closer to 12.0 cm the greater the involvement ( Figure 2B ).

After the end of all evaluations, the distance from the 0.0 cm point to the point marked on the scale by examiners was measured, in order to avoid interferences in classifications ( Figure 2C ). The intensity of atherosclerosis was classified as mild if the evaluation was from 0.1 cm to 4.0 cm; moderate, from 4.1cm to 7.0cm and accentuated, from 7.1cm to 12.0cm.^9^ Many degrees of atherosclerosis and accurate values were observed in mild, moderate and accentuated classification ( Figure 3 ).

**Figure 3:** – A) Atherosclerosis in aortic artery assessed as mild. B) Atherosclerosis in aortic artery assessed as moderate. C) Atherosclerosis in aortic artery assessed as accentuated.

### Statistical analysis

For statistical analysis, a database was created in the program *Microsoft Excel* ^®^. Data were analyzed using the *software GraphPad Prism* ^*®*^ 7.0. To verify the type of distribution of variables, the statistical test of *Kolmogorov-Smirnov* (with *Dallal-Wilkinson-Lillie* for p-value). For continuous variables with normal distribution, the mean and standard deviation were presented, and for those with non-normal distribution, the median and interquartile range. We used the unpaired student’s t-test (t) for normal distribution and the *Mann-Whitney* (U) for non-normal distribution comparison between two groups. For the comparison of three groups, Kruskal-Wallis (H) test was used, followed by Dunn post-test. Spearman’s correlation coefficient (rS) for non-normal distribution was used for correlation. A p-value less than 0.05 (typically ≤ 0.05) was considered statistically significant.

## Results

Regarding general distribution of the sample, data are described in Table 1 .

**Table 1:** – General characteristics of the sample

Variables	n (%)	Mean age (years ± SD)
**Total**	53 (100%)	49.9 ± 18.6
**Age**		
Elderly	17 (32.1%)	72.88 ± 8.1
Non elderly	3,983±2.59	39.05 ± 10.24
**Gender**		
Male	26 (49.1%)	49.77 ± 16.40
Female	27 (50.9%)	50.04 ± 20.77
Elderly male	7 (41.18%)	72 ± 6.35
Elderly female	10 (58.82%)	74 ± 9.42
Non elderly male	19 (52.78%)	41.58 ± 9.84
Non elderly female	17 (47.22%)	36.24 ± 10.22
**Cause of death**		
Cardiovascular	10 (18.9%)	64.3 ± 17.09
Non cardiovascular	43 (81.1%)	46.56 ± 17.43

*n: sample; SD: standard deviation.*

Carotid arteries showed greater atherosclerotic involvement than other arteries assessed (H = 15.73, p = 0.0004), with a significant difference found between carotid and iliac arteries (p = 0.0002). Variation in macroscopic degrees of atherosclerosis is described in Table 2 .

**Table 2:** – Macroscopic evaluation of right and left carotid, aorta and right and left iliac arteries of autopsied patients

Variables	Carotid (cm)	Aorta (cm)	Iliac (cm)
**n=53 (100%)**	**mean ±standard deviation or median (minimum-maximum)**		
Age			
Elderly	7,256±2,254	8.6 (1.3-9.8)	5.9 (1.2-9.07)
Non elderly	3,983±2.59	1.8 (0.5-3.6)	1.15 (0.1-2.5)
	t=6,321; p<0.0001	U=83.5; p<0.0001	U=306; p<0.0001
**Gender**			
Male	4,675±2,593	2.6 (0.5-10)	1.85 (0.1-10.8)
Female	5,378±3,178	3.7 (0.5-11.6)	2.85 (0.1-11.6)
	t=1,245; p=0.2160	U=285; p=2442	U=1165; p=0.1316
**Cause of death**			
Cardiovascular	7.7±2,617	7.25 (1.9-11.6)	5.1 (0.9-11)
Non cardiovascular	4,413±2,625	2.2 (0.5-11)	1.8 (0.1-11.6)
	t=5,047; p<0.0001	U=98.5; p=0.0068	U=467.5; p=0.0012

*n: sample; cm: centimeters.*

Macroscopic assessment and different degrees of macroscopic involvement of atherosclerosis in carotid, aortic and iliac arteries are shown in Figure 4 .

**Figure 4:** – A) Comparison of atherosclerosis macroscopic assessment in carotid, aortic and iliac arteries. B) Comparison between macroscopic evaluation of atherosclerosis in carotid, aortic and iliac arteries classified as mild (0 to 4 cm). C) Comparison between macroscopic assessment of atherosclerosis in carotid, aortic and iliac arteries classified as moderate (4.1 to 7 cm). D) Comparison between macroscopic assessment of atherosclerosis in the carotid, aortic and iliac arteries classified as accentuated (7.1 to 12 cm).

The occurrence of atherosclerosis was progressive and significant with age in carotid arteries (rS = 0.5133; p <0.0001), in aortic (rS = 0.716; p <0.0001) and in iliac arteries (rS = 0.7378; p <0.0001) ( Figure 5 ).

**Figure 5:** – A) Correlation between macroscopic evaluation of atherosclerosis in carotid arteries with age. B) Correlation between the macroscopic assessment of atherosclerosis in aortic arteries with age. C) Correlation between macroscopic assessment of atherosclerosis in iliac arteries with age.

Data regarding atherosclerosis macroscopic assessment and the variables analyzed are described in Table 2 .

## Discussion

Using autopsy material we demonstrated atherosclerosis in different arterial beds, which are of great importance for the body’s blood supply. Macroscopic analysis is a way to understand disease development process, being a valid and accurate instrument for research, providing data to clinical practice as already demonstrated in other studies.^9 - 11^

The present study demonstrated a greater involvement of carotid arteries, compared to aorta and iliac arteries, but with significant difference only between carotid and iliac arteries. Although atherosclerosis is a process that can affect the entire vascular tree, being found in any large or medium-sized arteries, the disease tends to be located in particular areas, such as aortoiliac, iliac-femoral segments or carotid arteries. Factors such as changes in blood flow, in extra vascular pressure and anatomical and biochemical features seem to explain the prevalence of lesions for these vessels.^12^ Furthermore, carotid arteries are evaluated in several other studies for atherosclerosis degree and determination of cardiovascular diseases development.^13 - 15^

In general, atherosclerosis degree varied between mild and moderate. In lesions evaluated as mild, fatty streaks were present, which indicates the beginning of injury process. Although these lesions do not alter blood circulation as they do not obstruct vascular lumen, their location facilitates continuous lipid deposition and progression to atherosclerosis.^16^

Elderly patients had significantly greater degree of atherosclerosis than non-elderly. Age was a significant predictor for atherosclerosis development.^17 , 18^ An increase in marked and asymptomatic atherosclerotic plaques was found in arteries from elderly patients,^19^ as well as calcifications,^20^ which corroborates our findings.

Regarding gender, a higher degree of atherosclerosis was found in women, but without significant difference, which agrees with a similar study that used ultrasound to analyze atherosclerosis, in which authors found no differences between genders.^21^ A recent study proved that genders have diferente physiological responses to risk factors (smoking, obesity, diabetes and systemic arterial hypertension) and females are the most affected and sensitized with such aggressions. Although many studies showed higher rates of cardiovascular events in men, there are divergent researches showing female physiological response is more sensitive to risk factors, which contributes to the development or worsening of cardiovascular disease.^22^

In the present study, patients who died due to cardiovascular causes had a significantly higher degree of atherosclerosis. The anatomopathological study of patients who died due to cardiovascular causes provides the best sample of population to study atherosclerosis.^4^ Cardiovascular diseases are directly associated with the occurrence of systemic atherosclerosis, asymptomatic in most cases,^23^ which makes prevention difficult, although extremely important.

As a *post-mortem* study, it has some limitations such as the absence of some data about patients’ lifestyle as medications, food, smoking, among other risk factors that are related to atherosclerosis development. In addition, some deaths occurred without atherosclerosis being previously investigated during patient’s hospitalization, which would be a good predictor for macroscopic evaluation accuracy. However, there are several positive points that strengthen the work, such as the direct and precise macroscopic evaluation through visualization of the entire plaque and the confirmation of association of intrinsic risk factors such as age and gender with cause of death that may have occurred due to extrinsic factors. In addition, several important arterial beds were collected (right and left carotid, right and left aorta and iliac) with similar results compared to risk factors and confirmed to be adequate sites for systemic atherosclerosis assessment.

## Conclusions

Atherosclerosis is a progressive lesion throughout life, which affects different arterial beds, with carotid arteries being the most affected, constituting an adequate sites for studying and assessing atherosclerotic plaque progression. The study highlights the importance of assessing atherosclerosis and shows an innovative form of assessment, as it is possible to measure the macroscopic degree of impairment through direct visualization of atherosclerotic plaques and compare with risk factors that, in association, can contribute to plaque progression and development of other cardiovascular diseases. Advanced age, female gender and death due to cardiovascular causes contribute as risk factors for greater lipid accumulation in these arteries. Macroscopic evaluation is a low-cost, effective and standardized method for measuring atherosclerosis degree and allows a better understanding of cardiovascular events development at the time of autopsy, in addition to providing data for clinical practice.
